# *In Vivo* Tracking of Murine Adipose Tissue-Derived Multipotent Adult Stem Cells and *Ex Vivo* Cross-Validation

**DOI:** 10.1155/2013/426961

**Published:** 2013-01-17

**Authors:** Chiara Garrovo, Natascha Bergamin, Dave Bates, Daniela Cesselli, Antonio Paolo Beltrami, Andrea Lorenzon, Roberto Ferrari, Carlo Alberto Beltrami, Vito Lorusso, Stefania Biffi

**Affiliations:** ^1^Optical Imaging Laboratory, Cluster in Biomedicine (CBM scrl), 34149 Trieste, Italy; ^2^Department of Cardiology, University of Ferrara, Salvatore Maugeri Foundation, IRCCS, 44100 Ferrara, Italy; ^3^VisualSonics Inc., Toronto, ON, Canada M4N 3N1; ^4^Centro Interdipartimentale di Medicina Rigenerativa, University of Udine, 33100 Udine, Italy; ^5^Ephoran Multi-Imaging Solutions, 10010 Colleretto Giacosa, Torino, Italy

## Abstract

Stem cells are characterized by the ability to renew themselves and to differentiate into specialized cell types, while stem cell therapy is believed to treat a number of different human diseases through either cell regeneration or paracrine effects. Herein, an *in vivo* and *ex vivo* near infrared time domain (NIR TD) optical imaging study was undertaken to evaluate the migratory ability of murine adipose tissue-derived multipotent adult stem cells [mAT-MASC] after intramuscular injection in mice. *In vivo* NIR TD optical imaging data analysis showed a migration of DiD-labelled mAT-MASC in the leg opposite the injection site, which was confirmed by a fibered confocal microendoscopy system. *Ex vivo* NIR TD optical imaging results showed a systemic distribution of labelled cells. Considering a potential microenvironmental contamination, a cross-validation study by multimodality approaches was followed: mAT-MASC were isolated from male mice expressing constitutively eGFP, which was detectable using techniques of immunofluorescence and qPCR. Y-chromosome positive cells, injected into wild-type female recipients, were detected by FISH. Cross-validation confirmed the data obtained by *in vivo*/*ex vivo* TD optical imaging analysis. In summary, our data demonstrates the usefulness of NIR TD optical imaging in tracking delivered cells, giving insights into the migratory properties of the injected cells.

## 1. Introduction

Optical imaging encompasses several desirable characteristics: it is rapid, noninvasive and nontoxic (it is not based on radiation). For these reasons, it is the optimal tool for performing long-term longitudinal studies *in vivo* [1], given the possibility to adapt experimental protocols to different fields of investigation. Specifically, time domain (TD) optical imaging technology allows for whole-body near infrared (NIR) fluorescence lifetime analysis, based both on the specificity of fluorescence probes and the sensitivity of their emission lifetime to environmental characteristics [2]. Different kinds of probes can be conjugated with fluorescence dyes: antibodies [3], polysaccharides [4], peptides [5], and also cells can be imaged to evaluate their *in vivo* biodistribution [6].

At present, clinical optical imaging is an emerging field, and its promising results are supported by preliminary investigations on sentinel lymph node tracers [7] and on peripheral tissue perfusion [8]. In both cases, indocyanine green was used as NIR fluorophore. Moreover, extensive studies have been conducted to confirm sensitivity and tissue diagnostic imaging potentiality of nanoparticles-based NIR contrast agents, such as quantum dots, resonant gold nanoshells, and dye-encapsulating nanoparticles [9]. 

In this study, we evaluate the applicability of the TD preclinical optical imaging system Optix to follow in the mouse the biodistribution of NIR-labelled murine adipose-tissue-derived stem cells (mAT-MASC). Stem cells are defined as undifferentiated cells able to both self-renew in the long-term and to differentiate into specialized cell types. Several studies have tried to dissect the mechanisms regulating the fate of stem cells residing in different tissues [10]. Skeletal muscle dystrophies comprise a heterogeneous group of neuromuscular disorders characterized by progressive muscle wasting [11], for which no satisfactory treatments exist [12]. Numerous different therapeutic strategies have been devised to correct the dystrophic phenotype [13]. In this regard, multiple stem cell populations, both of adult or embryonic origin, have been assayed for their myogenic ability. To date, many of these strategies have failed, thus, underlying the need to identify the mechanisms controlling myogenic potential, to avoid immune response, and to promote homing and engraftment of donor population to the musculature. In particular, it would be important to target lifesaving muscles, such as heart and diaphragm, which are involved in many dystrophies but are extremely difficult to access. 

Preliminary results showed that mAT-MASC were able, *in vitro*, to differentiate along myogenic lineages and, importantly, were characterized by a wide *in vivo* migratory ability, even when injected intramuscularly (i.m.). Nonetheless, in the present work we did not investigate how injected cells could undergo proliferation and differentiation into specific phenotypes, but we decided to optimize a multidisciplinary approach to evaluate the ability of TD optical imaging technology to monitor the *in vivo* distribution of i.m. injected stem cells. Specifically, male, DiD-labelled, and eGFP expressing mAT-MASC were delivered into a healthy, congenic, female recipient. Cell presence was evaluated, respectively, by *in vivo* NIR TD optical imaging and Cellvizio Lab dynamic fiberoptic fluorescence microscopy, by *ex vivo* TD optical imaging, qPCR, immunofluorescence associated with lambda-scan analysis, and FISH for Y-chromosome techniques. The results confirmed the potential of the applied complement of optical imaging techniques to be a complete and useful tool for tracking cell biodistribution and homing in small animals.

## 2. Materials and Methods

### 2.1. mAT-MASC Isolation and *In Vitro* Expansion

mAT-MASC were isolated from subcutaneous adipose tissues of 1- to 3-month-old, male C57BL/6-Tg[CAG-eGFP]1Osb/J[Jackson Laboratory] (*n* = 13) or wild-type C57BL/6 mice (*n* = 17) adapting the methods described previously [14, 15]. Briefly, subcutaneous adipose tissue harvested from mice was mechanoenzymatically dissociated using a digestion solution containing Joklik modified Eagle's medium and 0.05% collagenase type II (Sigma-Aldrich). After centrifugation at 900 g, cell suspension was filtered through a 70 *μ*m nylon membrane (Dako) and plated on fibronectin coated dishes (bovine origin, 10 *μ*g/100 mm plate, from Sigma-Aldrich) at the concentration of 4 × 10^4^ cells/mm^2^. Expansion medium was composed as follows: 60% low glucose DMEM, 40% MCDB-201, 1 mg/mL linoleic acid-BSA, 10^-9 ^M dexamethasone, 10^-4 ^M ascorbic acid-2 phosphate, 1x insulin-transferrin-sodium selenite (all from Sigma-Aldrich), 2% fetal bovine serum (StemCell Technologies) and 10 ng/mL mPDGF-BB and 10 ng/mL mEGF (both from Peprotech EC). The medium was replaced every 4 days. After 3 to 4 population doublings (corresponding to about 1 week in culture), cells were detached utilizing a 0.25% trypsin-EDTA solution (Sigma-Aldrich) and split onto fibronectin coated dishes at a density of 1-2 × 10^3^/cm^3^. Phase contrast images were captured utilizing an Olympus AX70 microscope connected to an Olympus DP50 camera (Olympus, Tokyo, Japan).

### 2.2. Flow Cytometry

Cells at the third passage in culture (P3) were detached and stained with the following properly conjugated antibodies: CD90, CD34, CD105, CD117, MHC-Ia/Ie, and MHC-1b (all from Santa Cruz Biotechnology), CD133, SCA-1, and CD9 (from eBioscience), CD44 and CD45 (from BD). Properly conjugated isotype matched antibodies were used as negative controls. The analysis was performed by CyAn (Beckman Coulter), and both, fraction of positive cells and intensity of expression, were evaluated for all antigens.

### 2.3. mAT-MASC Labelling with DiD

mAT-MASC cells were collected after trypsinization and suspended at a concentration of 1 × 10^6^/mL in serum-free culture medium. Following, 5 *μ*L of Vybrant DiD cell-labeling solution (Molecular Probes) was supplied per mL of cell suspension. DiD is a lipophilic carbocyanines tracer with markedly red-shifted fluorescence excitation and emission spectra which can be used for cellular adhesion studies and migration applications [16]. Once incorporated into membranes, DiD is highly fluorescent and photostable. The supplier reported high extinction coefficients (EC > 125,000 cm^−1^ M^−1^ at their longest-wavelength absorption maximum) and short excited-state lifetimes (~1 nanosecond) in lipid environments.

Cells were incubated for 1 hour at 37°C under agitation in the dark. Afterwards, the suspension was centrifuged at 900 g and washed three times with PBS to remove the free dye. At that time, DiD-labelled cells (1 × 10^6^/200 *μ*L serum-free culture medium) were seeded on an optical 96-wells-plate and analyzed with Optix in order to verify that the labelling process had been successfully succeeded.

### 2.4. Animal Model and Treatment for Optical Imaging Scan

Female C57/BL6 mice of 6–8 weeks old (*n* = 10) were purchased from Harlan (San Pietro al Natisone, Italy) and maintained under pathogen-free conditions. Mice were anesthetized using a gaseous anaesthesia system (Biological Instruments, Italy), based on isoflurane mixed to oxygen and nitrogen protoxide. Anaesthesia was first induced in a preanaesthesia chamber (2% isoflurane), and then the mouse was placed on the heated imaging bed of the Optix under 1% isoflurane. Moreover, mice were shaved in the regions of interest in order to avoid fur laser scattering. The experimental mice were injected in the left tibialis anterior muscle with 3 × 10^6^ mAT-MASC^eGFP^-DiD cells.

All the experimental procedures were conducted in compliance with the guidelines of European (86/609/EEC) and Italian (D.L.116/92) laws and were approved by the Italian Ministry of University and Research.

### 2.5. *In Vivo* Time Domain Optical Imaging Analysis

The *in vivo* TD small animal fluorescence imager Optix (ART Advanced Research Technologies Inc., Montreal, QC, Canada) was used in the study. 

In all imaging experiments, a 670 nm pulsed laser diode with a repetition frequency of 80 MHz and a time resolution of 12 ps light pulse was used for excitation. Fluorescence emission was collected at 700 nm and detected through a fast photomultiplier tube (PMT) coupled to a time-correlated single-photon counting system. Two-dimensional scanning regions of interest (ROI) were selected, and laser power, integration time, and scan step were optimized according to the emitted signal. The data were recorded as temporal point-spread functions, and the images were reconstructed as fluorescence intensity and fluorescence lifetime maps. All the *in vivo* analyses were preceded by native scans of the mice prior to injection of the labelled cells in order to provide a baseline for later analyses. Animals were followed for 48 hours (*n* = 4) or 7 days (*n* = 4) after the injection.

### 2.6. *Ex Vivo* Time Domain Optical Imaging Analysis

After the last imaging session was performed, mice were sacrificed by cervical dislocation in deep anaesthesia. The tissues of interest, such as the injected and the contralateral muscle, adipose tissues, brain, heart, diaphragm, liver, flexor digitorum brevis (FDB), spleen, and lung, were collected, washed with PBS twice, and imaged with the Optix system. 

### 2.7. Data Processing

To estimate fluorescence intensity and lifetime, the background signal intensity recorded with the baseline image for each animal before the injection of the labelled cells was subtracted from each postcontrast image using Optix OptiView software [17].

### 2.8. Fluorescence Intensity Analysis

Fluorescence intensity values are reported in normalized counts (NC) representing the photon count for unit excitation laser power and unit exposure time to allow comparison between different images. To perform the comparison, an identical ROI on each image was positioned to encompass the area under investigation. 

### 2.9. Fluorescence Lifetime Analysis

The fluorescence lifetime results are obtained by fitting every fluorescence decay curve corresponding to each pixel measured by Optix using the Levenberg Marquet least squares method [18].

### 2.10. *In Vivo* Dynamic Fluorescence Microscopy Cellvizio Lab

A probe-based confocal fluorescence microscope Cellvizio Lab (VisualSonics Inc. Toronto, Canada) was used. In all experiments the field of view was between 300 and 650 *μ*m, resolution from 1.4 to 3.5 *μ*m, the frame rate from 8 to 200 fr/sec, and excitation laser 680 nm.

Two healthy animals were treated i.m. with 3 × 10^6^ mAT-MASC^eGFP^-DiD in the left tibialis anterior muscle and analyzed by Cellvizio Lab 48 hours after the injection. Specifically, the day of the analysis mice were anaesthetized with 70 *μ*L of a solution 12% Zoletil 100 plus 7.5% Rompun 2% in Physiological saline solution i.m. to induce deep surgical anaesthesia and analgesia. Eyes were treated with an ophthalmic gel to prevent drying. Legs were shaved, and the area was disinfected with an iodopovidone solution. A small incision was made on the skin in the region of the thigh muscle with a 18G needle under sterile conditions, and the optical probe was inserted to record the images. At the end of the acquisition process, the incision was sutured with surgical and sterile silk.

### 2.11. Immunofluorescence and Confocal Microscopy

To perform immunolabelling assays, P3 mAT-MASC were fixed in 4% paraformaldehyde for 20 min at room temperature, permeabilized with 0.1% Triton X-100 (Sigma-Aldrich), and stained to visualize Oct4, Nanog, and Sox2 (Table 1).

Tissue specimens were collected either from mice injected with mAT-MASC^eGFP^-DiD or PBS. Excised tissues were partially formalin fixed and paraffin embedded and, in addition, part snap frozen. Immunofluorescence staining was performed on 5 *μ*m thick tissue sections. Primary antibodies, antigen retrieval, and staining protocols are listed in Table 1. Nuclei were stained by DAPI (Vector Laboratories, Inc), and Vectashield (Vector) was used as mounting medium. Epifluorescence and phase contrast images were obtained with a live cell imaging dedicated system consisting of a Leica DMI 6000B microscope connected to a Leica DFC350FX camera (Leica Microsystems); 10X (numerical aperture: 0.25), 40X oil immersion (numerical aperture: 1.25), and 63X oil immersion (numerical aperture: 1.40) objectives were employed. Confocal images were collected using Confocal Laser Microscope (Leica TCS-SP2, Leica Microsystems, Wetzlar, Germany), utilizing a 63X oil immersion objective (numerical aperture: 1.40) or a 40X oil immersion objective (numerical aperture: 1.25). Adobe Photoshop software was utilized to compose and overlay the images and to adjust contrast (Adobe, USA).

### 2.12. Fluorescence *In Situ* Hybridization (FISH)

Cambio's StarFISH Mouse Whole Chromosome-Specific paint kit was used to detect donor Y-chromosome in frozen sections. The probe was Cy3 labelled. Detection was performed following the protocol provided by the company. Sections obtained from female mice injected with PBS were employed as negative controls, while sensitivity was determined on sections obtained from untreated male mice.

### 2.13. Lambda Scan Analysis

This assay was performed using a Leica TCS-SP2 confocal microscope. Analyses were performed on murine sections (after appropriate immunolabelling) of mice injected with eGFP^+^DiD^−^ cells, eGFP^−^DiD^+^ cells, or eGFP^+^DiD^+^ cells. A555-labelled secondary antibody was utilized to recognize anti-eGFP primary antibody. The emission signal for Alexa555 was excited at 543 nm with an argon laser, and its fluorescence intensity was recorded generating a lambda stack ranging from 563 to 798 nm at 5 nm intervals. The emission signal for DiD was excited at 633 nm with a helium/neon laser, and its fluorescence intensity was recorded generating a lambda stack ranging from 653 to 798 nm at 6 nm intervals. The lens and corresponding numerical aperture were 63X and 1.4, respectively. Sampling consisted of 30 eGFP^+^ and/or DiD^+^ cells. Additionally, 10 cells negative for these markers and present in the same samples were used as control to discriminate background autofluorescence from specific labelling. Each determination was restricted to a region of interest (ROI) comprised within each DiD and/or eGFP-positive cell. For each ROI, a graph plotting mean pixel intensity and the emission wavelength of the lambda stack was generated. 

### 2.14. DNA Extraction and qPCR Analysis

Genomic DNA for PCR analysis was purified from frozen tissues using QIAmp DNA Mini Kit (QIAGEN). Weighed biopsies obtained from 2 mice injected with mAT-MASC^eGFP^-DiD, 2 untreated mice, and two C57BL/6-Tg[CAG-eGFP]1Osb/J[Jackson Laboratory] were incubated for 96 h at 56°C in lysis buffer and processed to purify the DNA, as recommended by the manufacturer. DNA concentration was estimated by using NanoDrop 2000 (Thermo Scientific), and quantitative real-time PCRs were performed using LightCycler 480 real-time PCR system (Roche). Amplification reactions were performed using manufacturer-provided reagents following the standard recommended amplification conditions (LightCycler 480 Probes Master). 50 ng of purified DNA from various tissues were amplified. Roche's Universal ProbeLibrary Assay platform was used to design primers and find suitable internal probes. The primers and probes for the target gene eGFP were forward primer 5′-GCATCGACTTCAAGGAGGAC-3′ and reverse primer 5′-GTTGATGTTGTCGGTGTTGCAG-3′; the probe labelled with fluorescent reporter and quencher was UPL PROBE#78 (Roche's Universal ProbeLibrary). As control, mouse beta-actin was amplified: forward primer 5′-CCATCTTGTCTTGCTTTCTTCA-3′ and reverse primer 5′-ATGAGACACACCTAGCCACC-3′; the probe was UPL PROBE#63 (Roche's Universal ProbeLibrary). To determine amplification efficiency and to estimate, for each specimen, the absolute concentration of both eGFP gene and beta-actin gene, calibration curves for eGFP gene and beta-actin gene, respectively, were constructed. Negative control for the target gene was isolated from mice that did not undergo transplantation. Positive control DNA was isolated from C57BL/6-Tg[CAG-eGFP]1Osb/J[Jackson Laboratory]. To quantify the number of transplanted eGFP cells in mouse tissues, we calculated the ratio between beta-actin and eGFP copy numbers and multiplied this value per 100000. Therefore, we could express the ratio of eGFP DNA copy numbers per 100000 murine cells.

## 3. Results 

### 3.1. Multipotent Adult Stem Cells Were Isolated from Mouse-Derived Adipose Tissue and Characterized

**Figure 1:**
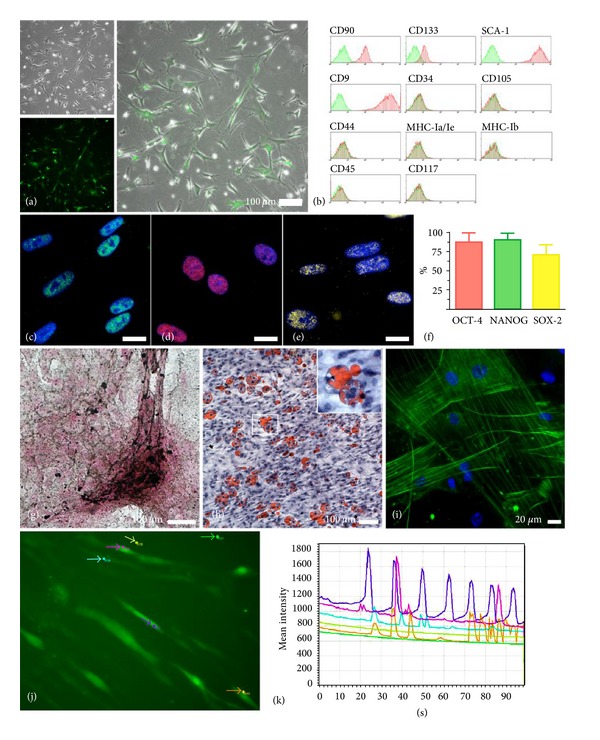
mAT-MASC^eGFP^ characterization. (a) Phase contrast (upper left) and eGFP fluorescence (lower left) images of mAT-MASC^eGFP^ at the third passage in culture. The right panel represents the overlapping of the previous ones (calibration bar 100 *μ*m). (b) Surface immunophenotype: representative flow cytometry histograms of mAT-MASC. Plots show isotype control IgG-staining profile (green histogram) versus specific antibody staining (red histogram). (c–f) Pluripotent state-specific transcription factor expression: Oct-4 (green fluorescence; (c)), Nanog (red fluorescence; (d)), and Sox-2 expression (yellow fluorescence; (e)) in the nuclei of mAT-MASC. Nuclei are depicted by the blue fluorescence of DAPI staining ((c–e), calibration bars 20 *μ*m). (f) Quantification of pluripotent state-specific transcription factor expression. Data are presented as mean ± standard deviation. (g–k) Multipotency of mAT-MASC^eGFP^. mAT-MASC cultured in osteogenic medium display positivity for von Kossa (brown deposits; (g), calibration bar 100 *μ*m), while cultured in adipogenic medium acquired positivity for Oil-Red-O (red deposits; (h), calibration bar 100 *μ*m). A fraction of mAT-MASC^eGFP^ in myogenic medium expressed the myocyte-specific filament SMA (green fluorescence, (i), calibration bar 20 calibration bar 100 *μ*m) and displayed spontaneous calcium transients (j-k). Specifically, cells loaded with Fluo-4 emitted green fluorescence in response to intracytoplasm calcium release (green fluorescence; (j)). In the graph (k), each colored line represents the changes of fluorescence intensity with time within the ROI identified in Figure (k) by the same color.

In order to obtain mAT-MASC (i.e., multipotent adult stem cells from murine adipose tissue samples) we applied, with minor modifications, the method previously described for the isolation of human MASC from liver, heart, bone marrow [14], and peripheral blood [15]. The isolation protocol allowed establishment and *in vitro* expansion of cell lines, named mAT-MASCs, from adipose tissue fragments obtained both from mice constitutively expressing eGFP [C57BL/6-Tg[CAG-eGFP]1Osb/J[Jackson Laboratory] (*n* = 13) and C57BL/6 wild-type mice (*n* = 17). mAT-MASC were obtained from all samples, confirming the high reproducibility and efficiency of the optimized method [14, 15]. All cell lines were grown on fibronectin coated dishes, in an expansion medium supplemented with mPDGF-BB, mEGF, and containing 2% FBS. Primary cultures reached 80% of confluence within one week, and, at the third passage in culture (P3, 20th-25th generation), mAT-MASC displayed a homogeneous fibroblast-like morphology (Figure 1(a)) and had a population doubling time of 33 ± 6 hours. As expected, mAT-MASC obtained from transgenic mice (mAT-MASC^eGFP^) showed a variable expression of eGFP protein (Figure 1(a)). The surface immunophenotype of wild-type and mAT-MASC^eGFP^-DiD was assessed by flow cytometry at the third passage in culture (*n* = 5). All cell lines shared a similar mesenchymal immunophenotype characterized by a larger fraction of cells expressing CD90, CD9, CD13, and Sca-1, while CD45, CD117, CD105, CD34, MHC-I, MHC-II, and CD44 were expressed in a minority of the cells (Figure 1(b)). This antigenic pattern was very similar to the one described by Verfaillie's group for murine MAPCs [19]. To evaluate if adipose-derived cell lines displayed stem cell properties, we tested the expression of transcription factors known to be associated with a pluripotent state [20]. As shown in Figures 1(c)–1(f), Oct-4, Nanog, and Sox-2 proteins were detected, by immunofluorescence, in a large fraction of mAT-MASC at the third passage in culture, confirming the undifferentiated state of the cultured cells. In order to evaluate whether mAT-MASC were characterized by the ability to differentiate into multiple mature cell types of mesodermic origin, cells were cultured under appropriate differentiation inducing conditions (see Section 2), and the acquisition of functional as well as molecular evidence of differentiation was assessed. Cells cultured in an osteogenic medium exhibited calcium deposits (Von Kossa staining, Figure 1(g)), while the capability of mAT-MASC to differentiate into adipocytes was demonstrated by their ability to store neutral triglycerides and lipids (Oil Red-O staining, Figure 1(h)). The ability of mAT-MASC to differentiate into muscle cells was tested in a medium containing VEGF, bFGF, and IGF-1. After the differentiation period, a fraction of cells, lower than 30%, expressed organized filaments of smooth-muscle actin (SMA) (Figure 1(i)), while almost all cells showed organized filaments of alpha-sarcomeric actin (ASA) (data not shown). The presence of functional competent receptors involved in calcium handling was demonstrated by spontaneous intracellular calcium transients, as displayed by Fluo-4 assays (Figures 1(j)-1(k)). Altogether, the accumulated evidence shows that mAT-MASC represent a population of primitive, multipotent cells easy to obtain and expand *in vivo* and, therefore, they are a suitable cell source for *in vivo* regenerative study. 

### 3.2. mAT-MASC Cells Implantation Can Be Tracked with a NIR Agent by *In Vivo* TD Optical Imaging

**Figure 2:**
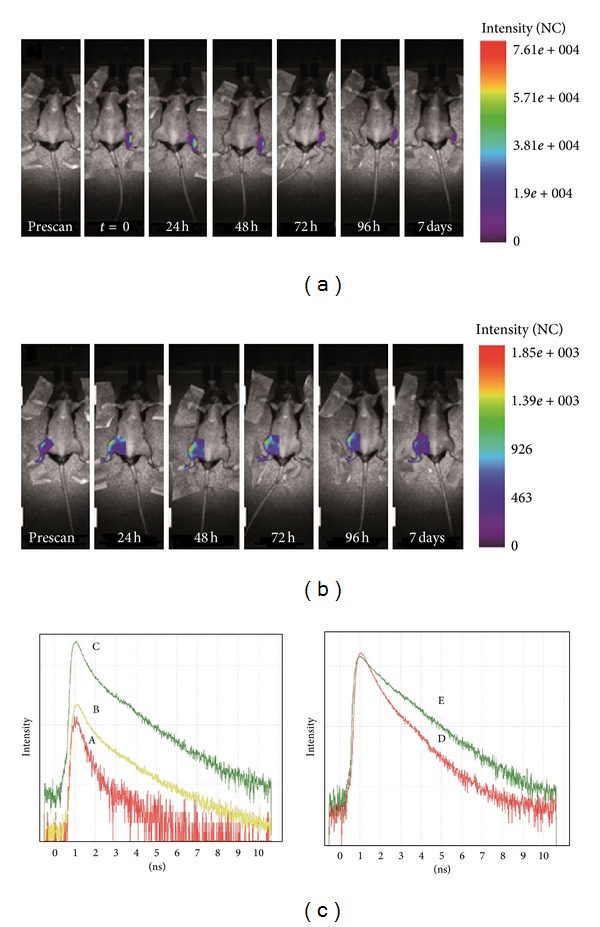
*In vivo* fluorescence intensity analysis over time and whole body scan biodistribution of 3 × 10^6^ mAT-MASC^eGFP^ labelled with DiD in a healthy animal model after i.m. injection in the left tibialis anterior muscle (a). *In vivo* fluorescence intensity analysis of contralateral (and not injected) leg. The scans were optimized in this region to visualize changes in terms of fluorescence intensity. The peak is reached 48 hours after the injection of the labelled cells (b). (c) *In vivo* lifetime analysis and comparison between the control (A), the contralateral leg 48 hours after mAT-MASC^eGFP^-DiD i.m. injection (B), and the injected muscle (C). (B) and (C) have comparable trends and comparable value (see Table 2). (c) *In vitro* lifetime analysis of mAT-MASC^eGFP^-DiD (D) and free DiD (E): the different trend of the curves indicates the possibility to discriminate by a lifetime analysis between the labelled cells and the pure dye (for the values see Table 2).

In order to track mAT-MASC after implantation, an NIR agent for cells labelling with TD optical imaging was applied. Cells labelled with DiD were injected i.m. into the left tibialis anterior muscle of healthy mice. Whole body scans showed the highest fluorescence signal in the region of injection, while intensity decreased exponentially over time according to the cells migration (Figure 2(a)). In order to optimize fluorescence acquisition parameters, the cells injection region was maintained outside the scanning area, and a manually selected region of interest (ROI), encompassing the contralateral leg, was investigated. The fluorescence signal increased slowly and was significantly higher than precontrast from 24 hours until 7 days after injection (Figure 2(b)), with a maximum at 48 hours. At this time point, we compared temporal point spread function (TPSF) curves resulting from prescan and scan after injection. As shown in Figure 2(c), *in vivo* labelled cells emitted a fluorescence intensity characterized by a specific TPSF curve (Figure 2(c), C), comparable with TPSF curves resulting from contralateral leg analysis (Figure 2(c), B) and different from the one obtained from the control (Figure 2(c), A). In addition, analysis performed *in vitro* on mAT-MASC-DiD and free DiD showed two different lifetimes with different values (Figure 2(c), D, and E). This result provided the possibility to discriminate between the labelled cells and the free dye, in order to confirm that the signal under investigation *in vivo* was not amenable to DiD itself. The summary of lifetime is reported in Table 2: to compare the data, all the values were acquired in a normalized range between 3 and 9 ns.

### 3.3. *Ex vivo* TD Optical Imaging Confirmed the *In Vivo* Results

At the end of the experiments, mice were sacrificed by cervical dislocation, and the organs were excised and washed in PBS. Then, legs muscle, brain, heart, diaphragm, liver, FDBs, spleen and lung were analyzed by Optix optical imaging. This way, any unspecific contribution due to autofluorescence from other organs and absorption and scattering within the body and fur could be avoided, thereby increasing both specificity and sensitivity of the probe detection.

**Figure 3:**
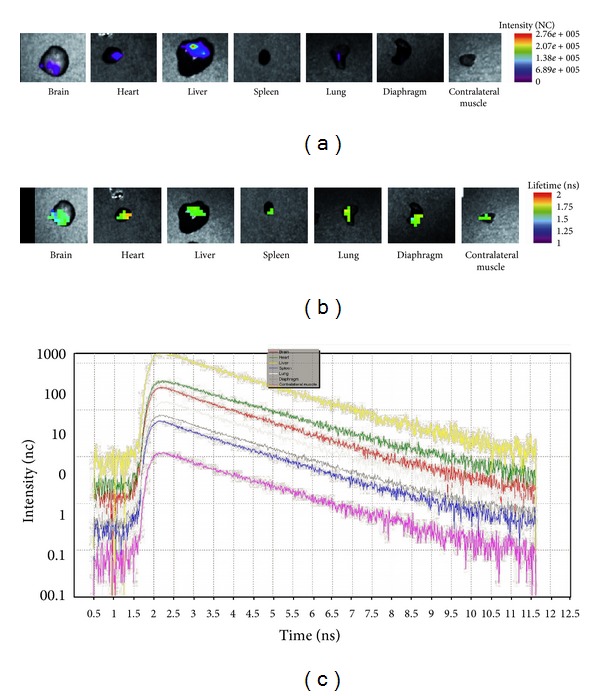
(a) *Ex vivo* fluorescence intensity analysis 48 hours after i.m. mAT-MASC-DiD injection. Mice were sacrificed, and organs were excided and analyzed by Optix. The fluorescence intensity map is shown. (b) Applying a lifetime gate (1.3–1.8 ns, corresponding to the lifetime range of DiD) to the initial intensity images yielded a map of mAT-MASC^eGFP^-DiD biodistribution and eliminated background signal. (c) The graph representing lifetime trends confirms the comparability between the signals recorded from the different organs *ex vivo*, which are represented by the same slope of the curves. The colors, respectively, represent yellow, liver; green, heart; red, brain; white, lung; grey, diaphragm; blue, spleen; pink, contralateral muscle.

*Ex vivo* evaluation of organs at 48 hours after injection clearly showed that the highest fluorescence emission was detected in the liver (Figure 3(a)): considering this short interval of time, 48 hours, we cannot exclude the possibility that cells managed a method of systemic distribution. Applying a lifetime gate corresponding to the lifetime range of DiD-labelled cells (1.3–1.8 ns) to the initial fluorescence intensity, images yielded a map of mAT-MASC-DiD biodistribution and eliminated background signal. Lifetime gating confirmed the specificity of the signals with a common mean value of 1,6 ns (Figure 3(b)), and lifetime curves analysis reported comparable trends for all the excised tissues (Figure 3(c)), 

The analysis performed in the animals sacrificed one week after the cell injection showed the presence of a specific signal only in the injected muscle and in the surrounding fat, but not in the other analyzed tissues (data not shown). The result suggested the hypothesis that, in this interval, mAT-MASC-DiD are probably spread in the body in a concentration too low to be detectable by Optix. Only in the region of injection, which was characterized by a high starting concentration, there is a weak but detectable signal.

Finally, the *in vivo* and *ex vivo* optical imaging procedures here previously described, which were optimized to monitor cells migration, are purely qualitative and not quantitative, and the figures reported are representative. For this reason, no intersubject variation relative to the migration of labelled cells has been performed.

### 3.4. mAT-MASC-DiD Cells Migration Can Be Visualized by *In Vivo* Dynamic Fluorescence Microscopy Cellvizio Lab

**Figure 4:**
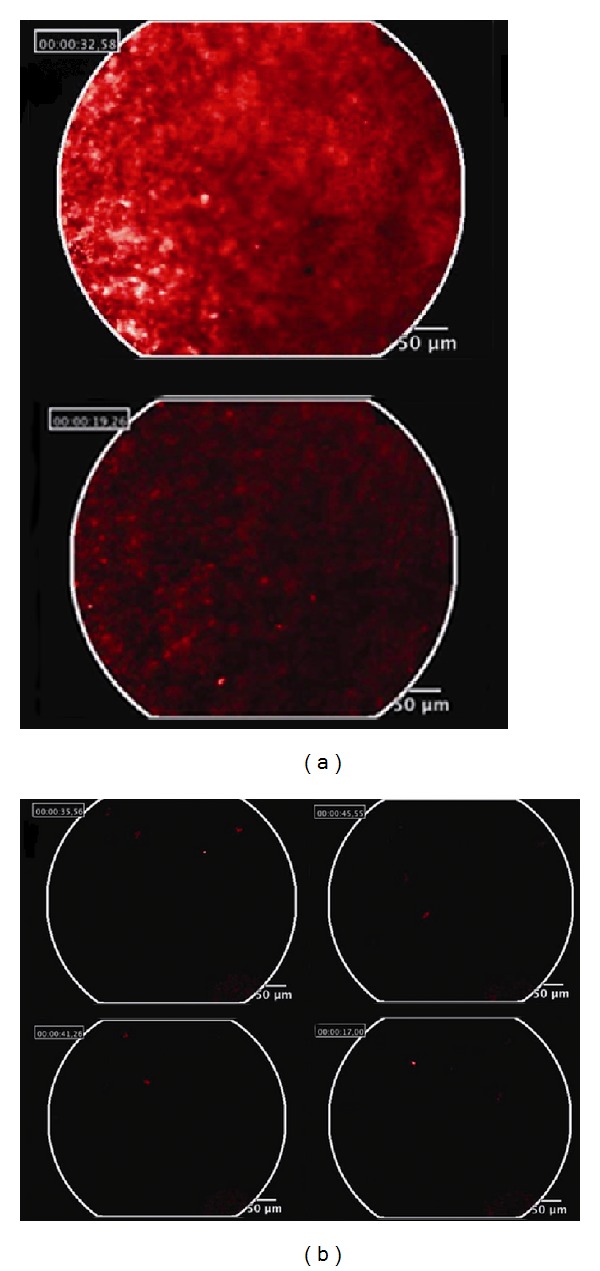
*In vivo* dynamic fluorescence microscopy Cellvizio Lab. The abundance of mAT-MASC-DiD cells in the left injected leg (a) is visualized. It was also confirmed cells' migration from the injection site by means of individual cells' tracking in the contralateral leg (b). For more details see Video1 and Video2 in the Supplementary Materials.

The application of Cellvizio Lab fiberoptic fluorescence microscopy allowed us to collect longitudinal high resolution data with a low invasiveness in living animals. It was possible to study cell homing and migration in real-time *in vivo* 48 hours after cells injection. The results obtained confirm that in the left injected leg there was an abundance of mAT-MASC^eGFP^-DiD cells (Figure 4(a)) (see Video 1, Supplementary Material available online at http://dx.doi.org/10.1155/2013/426961) and that in the right contralateral leg it was feasible to image individual cells which had migrated from the injection site (Figure 4(b)) (Video 2, Supplementary Material). Finally, while individual cells could not be imaged by means of Optix system, *in vivo* fiberoptic fluorescence microscopy allowed us to track single cells that were heterogeneously dispersed, confirming their ability to migrate *in vivo*. 

### 3.5. qPCR Analysis Allowed to Estimate the mAT-MASC^eGFP^-DiD Cells Biodistribution

**Figure 5:**
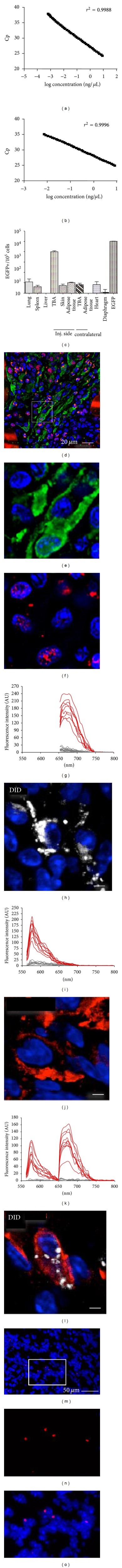
mAT-MASC^eGFP^-DiD distribution and characterization. (a, b, c) Real-time PCR analysis was performed to detect transgenic cells from recipient mice. Calibration curves, respectively, for the gene eGFP (a) and beta-actin (b) were produced to extrapolate the absolute concentration of both genes for each analyzed tissue. In (c) the relative amount of eGFP cells per 10^5^ nuclei detected in lung, spleen, liver, injected tibialis anterior muscle (TA Inj.), adipose tissue and skin near the site of injection (ADIP. Inj., and SKIN Inj.), diaphragm, heart, contralateral adipose tissue (ADIP. Contral.), and tibialis anterior muscle were reported. eGFP column indicated a representative value obtained from eGFP transgenic tissue. mAT-MASC^eGFP^ presence 7 days after cell delivery was confirmed by immunofluorescence assays (d, e), green fluorescence (calibration bar 20 *μ*m) (j, l) red fluorescence, calibration bar 20 (*μ*m). Lambda scan analysis was performed to confirm the specificity of the signal. Emission spectra for DiD (g) (red line) and eGFP, Alexa 555 (i) (red line) were singularly analyzed and appeared distinct from the tissue autofluorescence emission spectra (g, i) gray lines. (k) Cells exhibiting at the same time spectra specific for DiD emission and eGFP (Alexa 555 emission) were found at the injected site (red line) and not in tissue sections obtained from not-injected mice (grey lines). Detection of male mAT-MASC^eGFP^ by FISH analysis (m, n, o). The presence of Y-chromosome positive cells (red spots) in female tissues suggested that viable donor cells persisted in tibialis anterior muscle (calibration bar 50 *μ*m). (d, e, f) By costaining cells for eGFP (green fluorescence) and MCM5 (red fluorescence) double positive cells were detected, indicating that injected cells were viable and proliferated.

To extend data obtained from Optical Imaging analysis and to quantify the amount of engrafted cells 7 days after injection, quantitative real-time PCR was performed. Genomic DNA was extracted from frozen tissues of mice injected with 3 × 10^6^ mAT-MASC^eGFP^-DiD, not injected mice (negative control), and transgenic mice constitutively expressing eGFP (positive control). To evaluate the efficiency and to calculate the regression *r* value, serial dilutions of genomic DNA extracted from eGFP^+^ and wild type mice were used to create distinct standard curves (Figures 5(a) and 5(b)). Absolute concentration of both, eGFP and *beta actin* gene, was extrapolated from each standard curve (Figure 5(c)). The relative amount of eGFP expressing cells in each of the evaluated tissues was estimated as a ratio of eGFP and *beta actin* gene copies and normalized to 100,000 nuclei (Figure 5(c)). As reported in the graph, only a small number of mAT-MASC^eGFP^-DiD cells could be detected 7 days after cell injection into the major filter organs like the spleen and the lung (between 1/and 10/100,000 total cells), but never in the liver. As expected, the injected muscle showed the highest mAT-MASC^eGFP^-DiD cell content (10,000 ± 1,361 eGFP^+^ cells/100,000 total cells). Moreover, we confirmed the presence of eGFP^+^ cells in tissues far from the injection site, like the contralateral tibialis anterior muscle, indicating that cells could migrate and persist in tissues other than the injection site for at least 7 days, even if no pathological conditions were induced in the recipient animal. Of great interest, both diaphragm and heart contained eGFP^+^ cells (Figure 5(c)). This is of paramount importance, considering that both organs can be severely involved in muscular dystrophies [21] and they are notoriously difficult to reach for cell-based therapy techniques [13]. 

### 3.6. *In Situ* Identification and Characterization of mAT-MASC^eGFP^-DiD into the Injection Site

With the aim of confirming the presence of and to localize mAT-MASC^eGFP^-DiD in situ, two independent techniques were adopted: (1) eGFP detection by immunofluorescence in association with lambda scan analysis and (2) fluorescence *in situ* hybridization (FISH) specific to the murine Y chromosome. The *in situ* presence of mAT-MASC^eGFP^ was further demonstrated by using an antibody recognizing the endogenous expression of the eGFP protein (Figures 5(d)–5(e)). To validate the specificity of the recorded signals, spectral analysis was performed [22]. The emission spectra for GFP and DiD were clearly distinct from the emission spectra for tissue autofluorescence, confirming the accuracy of the immunolabelling protocol (Figures 5(g), 5(i), and 5(k)). 

As shown in panel k and l, cells simultaneously displaying spectra resembling the single emissions of DiD or Alexa 555 (fluorophore conjugated to the secondary antibody developed against specific eGFP primary antibody) were specifically found in injected muscle (red lines) and not in tissue sections obtained from not-transplanted mice (grey lines). 

Since male mAT-MASC^eGFP^-DiD were i.m. injected into female recipients, the presence, in injected female muscle sections, of Y-chromosome positive cells supported the evidence that viable donor cells persisted in tibialis anterior muscle (Figures 5(m)–5(o)). 

Vitality and proliferative rate acquired by donor cells *in vivo* were further assessed by costaining cells for eGFP and MCM5 (Figures 5(d)–5(f)), a protein involved in the control of DNA replication. We estimated that 51 ± 5.7% of eGFP-positive cells were positive for the proliferation marker suggesting that engrafted cells retain the ability to duplicate *in vivo*. 

## 4. Discussion 

Here, NIR TD optical imaging technology for *in vivo* longitudinal studies based on NIR dye labelled-cells and fluorescent lifetime analysis was applied, and these characteristics enable both high specificity and sensitivity for biodistribution studies due to the dyes' spectral characteristics [1, 2]. Specifically, we evaluated the migratory ability of mAT-MASC injected i.m. in mice. mAT-MASC were cultured utilizing methods previously optimized to expand multipotent adult stem cells [14, 15]. 

To investigate whether TD optical imaging could track *in vivo* mAT-MASC, 3 × 10^6^ DiD-labelled cells were injected into the left tibialis anterior muscle of a congenic wild-type female recipient. Animals were monitored for a short (48 hours) time and also for an extended period (7 days) to evaluate cells biodistribution over time. Injected animals that were analyzed within the first 48 hours after injection by TD optical imaging showed a specific signal in the region of the injection that decreased with time. Conversely, the fluorescence intensity analysis performed in the contralateral leg revealed a slow increase with a peak signal after 48 hours. To assess the cell migration and to see if the signal can be discriminated from the free dye, we performed lifetime analysis, based on the comparison between lifetime values obtained for the injection site and the contralateral leg. The relative curves obtained showed similar trends and comparable values (1.50 and 1.56 ns, resp.). Moreover, it can be assumed that the signal is not due to the free dye because it was demonstrated that labelled cells and DiD have different specific lifetime values (1.55 and 1.10 ns resp.).

*Ex vivo* analysis performed 48 hours after the injection confirmed the data obtained *in vivo*, and in addition it revealed the presence of a specific signal in filter organs such as the lung, spleen, and liver, as well as the brain, adipose tissue, left and right flexor digitorum brevis, diaphragm and heart. This homogeneous presence of mAT-MASC in all the organs analyzed could be traced to the nonspecific biodistribution of the cells in the short period that had elapsed after treatment. *Ex vivo* analysis and *in vivo* TPSF curve evaluation suggested the presence of mAT-MASC^eGFP^-DiD in the leg opposite the injection site, supporting the hypothesis that the cells reached, possibly through systemic distribution, distant sites.

To further investigate and demonstrate the specificity of the signal cell migration was analysed 48 hours following injection by a fibered confocal microendoscopy system, which provided a direct, rapid, and accurate visualization of labelled cells at the muscle in both legs. 

In order to evaluate whether mAT-MASC^eGFP^-DiD could persist longer, a new group of animals was analysed daily by Optix preclinical optical imager for 7 days after the injection. A progressive decrease in fluorescence signal in the region of the treatment was observed, and at one week, no specific signal could be detected in the leg opposite the injection site. It could be supposed that one week after the treatment the cells are too dispersed throughout the body or that the dye is too diluted to be detected by Optix. This result gave a complete overview of how much the experiment can be projected into a longitudinal scheme. 

At the same time point of one week after cells injection, the *in vivo* results have been confirmed by *ex vivo* optical imaging analysis where no specific signal had been detected neither analysing the organs singly, except for the injected muscle.

Considering a potential microenvironmental contamination by cell-free DiD, tracking of labelled cells using nongenetically encoded markers should always be accompanied by cross-validation using multimodality approaches [21]; therefore, we used cells that could be recognized by different *in vivo* and *ex vivo* techniques. Specifically, mAT-MASC were isolated from male mice expressing constitutively eGFP as marker gene product, which was readily detectable using techniques of immunofluorescence (eGFP protein) and qPCR (eGFP gene). Moreover, male cells injected into wild-type female recipients were identified by FISH detection of the Y-chromosome. An assay aimed at detecting both eGFP and beta-actin genes was optimised, thus allowing quantification of the relative number of eGFP^+^ cells into wild type tissues. As expected, the highest number of eGFP^+^ cells was detected at the injection site, where 10% of the nuclei were estimated to harbour the transgene. In all the other analyzed tissues the estimated number of eGFP^+^ nuclei was extremely low, between 1 and 10 cells per 10^5^ total nuclei. Engrafted cells found at the site of implantation were viable and proliferating, and almost half of them were differentiated into myocytes and CD146 positive vascular cells (data not shown). Despite the fact that lifetime analysis is able to distinguish DiD-labelled cells with high specificity, the sensitivity of the technique does not allow detection of very rare cells (1–10 eGFP^+^/10^5^ nuclei); therefore additional techniques must be applied with this purpose.

## 5. Conclusion 

In the present work, the results showed the utility of using an imaging technologies panel to track delivered cells, especially during the first days after injection, giving insights into their migratory properties. Cells biology and differentiation capability were not the specific goals of this paper: the experiments have been originally planned to define an appropriate tool to obtain a cross-validated study, both *in vivo*, for analyzing cells biodistribution and their specific localization, and *ex vivo*, for evaluating their accumulation and properties. In particular, knowledge about the biodistribution of mAT-MASC was acquired: such as confirmation of migratory properties and, at the same time, monitoring them using different imaging applications, long and short times after injection for evaluation.

It can be concluded that the multimodality approach we applied could be of paramount importance in the study of systemic diseases, where it could be utilized to monitor the effect of immunomodulatory or other pharmacological interventions on engrafted cell number and distribution. Finally, the proven ability of mAT-MASC^eGFP^-DiD to migrate to distant targets suggests that they could represent a suitable cell source to be utilized in systemic diseases, such as the skeletal muscle disorders, and that the future perspectives would be to further investigate mAT-MASC behaviour, proliferation, and differentiation* in vivo.*

## Supplementary Material

The supplementary material consists of a video reproducing the two results obtained with Cellvizio LAB. The video shows the real-time images recorded with the fiberoptic fluorescence microscopy in a mouse injected 48 hours before with labelled mAT-MASC. The analysis has been performed both in the injected leg (Video 1) and in the contralateral one (Video 2).

## Figures and Tables

**Table 1:** Cell and tissue staining protocols.

Primary antibody				Secondary antibody		
Antigen	Producer	Antigen retrieval	Dilution	Fluorochrome	Producer	Dilution
OCT4	ABCAM	TRITONX-100 0.1%	1 : 200	A488	Molecular probes	1 : 400
NANOG	ABCAM	TRITONX-100 0.1%	1 : 400	A488	Molecular probes	1 : 400
SOX2	ABCAM	TRITONX-100 0.1%	1 : 200	A488	Molecular probes	1 : 400
eGFP	ABCAM	TRITONX-100 0.1%	1 : 80	A488 A555	Molecular probes	1 : 400 1 : 600
MCM5	ABCAM ABCAM	TRITONX-100 0.1%	1 : 80 1 : 100	A555 A488	Molecular probes	1 : 600 1 : 400
eGFP MMP2	ABCAM ABCAM	TRITONX-100 0.1%	1 : 80 1 : 80	A488 A555	Molecular probes	1 : 400 1 : 600
eGFP ASA	ABCAM Sigma	TRITONX-100 0.1%	1 : 80 1 : 100	A488 TRITC [IgM]	Molecular probes Jackson ImmunoResearch	1 : 400 1 : 100
eGFP CD146	ABCAM ABCAM	TRITONX-100 0.1%	1 : 80 1 : 20	A488 A555	Molecular probes	1 : 400 1 : 600
eGFP SMA	ABCAM DAKO	TRITONX-100 0.1%	1 : 80 1 : 40	A555 A488	Molecular probes	1 : 400 1 : 600
Laminin ASA	DAKO Sigma	TRITONX-100 0.1%	1 : 50 1 : 100	A488 Cy5 [IgM] A555	Molecular probes Jackson ImmunoResearch	1 : 400 1 : 100 1 : 600

**Table 2:** Lifetime values summary.

	Lifetime [ns]
*In vivo* control [a]	1.26
*In vivo* contralateral leg t48h [b]	1.56
*In vivo* injected leg t48h [c]	1.50
*In vitro* labelled cells [d]	1.55
*In vitro* DiD [e]	1.10

## Abbreviations

mAT-MASC: Murine adipose tissue-derived multipotent adult stem cells
i.m.: Intramuscular
NC: Normalized counts
NIR: Near infrared
TD: Time domain
ROI: Region of interest
qPCR: Quantitative PCR
TPSF: Temporal point spread function
ns: Nanoseconds.
